# SPOTONE: Hot Spots on Protein Complexes with Extremely Randomized Trees via Sequence-Only Features

**DOI:** 10.3390/ijms21197281

**Published:** 2020-10-01

**Authors:** A. J. Preto, Irina S. Moreira

**Affiliations:** 1 CNC—Center for Neuroscience and Cell Biology, University of Coimbra, 3004-504 Coimbra, Portugal; martinsgomes.jose@gmail.com; 2 Department of Life Sciences, Center for Neuroscience and Cell Biology, Coimbra University, 3000-456 Coimbra, Portugal

**Keywords:** big-data, hot-spots, machine learning, protein–protein complexes, structural biology

## Abstract

Protein Hot-Spots (HS) are experimentally determined amino acids, key to small ligand binding and tend to be structural landmarks on protein–protein interactions. As such, they were extensively approached by structure-based Machine Learning (ML) prediction methods. However, the availability of a much larger array of protein sequences in comparison to determined tree-dimensional structures indicates that a sequence-based HS predictor has the potential to be more useful for the scientific community. Herein, we present SPOTONE, a new ML predictor able to accurately classify protein HS via sequence-only features. This algorithm shows accuracy, AUROC, precision, recall and F1-score of 0.82, 0.83, 0.91, 0.82 and 0.85, respectively, on an independent testing set. The algorithm is deployed within a free-to-use webserver, only requiring the user to submit a FASTA file with one or more protein sequences.

## 1. Introduction

Hot-Spots (HS) can be defined as amino acid residues that upon alanine mutation generate a change in binding free energy (ΔΔG_binding_) higher than 2.0 kcal mol^−1^, in opposition to Null-Spots (NS), which are unable to meet this threshold. Although the threshold of 2.0 kcal mol^−1^ can vary in the definition of HS, a representative amount of studies on the subject typically use this cut-off [1,2,3,4,5,6]. HS are key elements in Protein–Protein Interactions (PPIs) and, as such, fundamental for a variety of biochemical functions. The disruption of these interactions can alter entire pathways and is of interest to therapeutic approaches [1,7]. These residues are also known to be important for protein dimerization [8] and ligand binding [9]. Indeed, HS tend to be associated with the binding of small ligands, hence becoming ideal subjects of study on target proteins for drug design approaches [9,10,11].

Databases of experimental determined HS and NS can be found in the literature: ASEdb [12], BID [13], PIN [14] and SKEMPI [15]. More recently, SKEMPI 2.0 was released, making available a larger amount of experimental information. However, most of the new information does not include mutations to alanine (and the corresponding change in free binding energy), which is the material under scope in the present work [16]. These databases can be used to deploy Machine-Learning (ML) algorithms that take both the positive (HS) and negative (NS) information and construct a binary classifier that should be able to predict, upon previously unforeseen amino acid residues in a protein, its HS/NS status. Although ML is not limited to binary classification, on this problem and given the available data format, binary classification was the most explored approach until now. Several algorithms have been proposed for HS computational predictions, using different ML approaches, features and datasets [17,18,19,20,21,22,23,24,25]. Recently (2017), SPOTON [22], using information on both the protein sequence and structure, achieved results of 0.95 accuracy on an independent testing set, making it the best performing HS predictor at the time. Most of the high-performing HS predictors incorporate structural information. Although yielding clearly robust results, it hinders the possibilities of a broader deployment, since there are still fewer proteins for which a three-dimensional (3D) structure is available in online repositories [26] compared to the determined and available protein sequences [27]. It is known that sequence-based predictors tend to perform more poorly, in comparison with the ones engulfing structural information. For example, Nguyen et al. (2013) [19] were able to achieve an accuracy of 0.79 and a precision of 0.75 using sequence-based frequency-derived features. More recently, Hu et al. (2017) [20] achieved an F1-score of 0.80 using only sequence-based features while Liu et al. (2018) [21] achieved an F1-score of 0.86 using sequence-based features and amino acid relative Solvent Accessible Surface Area (SASA). The problem of HS computational determination is usually riddled with class imbalance, as there are commonly more experimentally determine residues as NS than HS due to the nature of PPIs. Conversely, the size of the dataset is usually not large enough to dilute this discrepancy. As such, problems emerge on the dataset training, but, more importantly, on the analysis of the results. We developed SPOTONE (hot SPOTs ON protein complexes with Extremely randomized trees), a HS predictor that only makes use of protein sequence-based features, all of which were calculated with an in-house Python pipeline. To avoid protein-centered overfitting, features concerning the whole protein were not applied to the classification problem. This allowed us to avoid the predictor from learning HS/NS only on a specific subset of proteins and be able to correctly classify even for unforeseen subtypes of biological machineries. Furthermore, we deployed a rigorous train–test split that ensured equality among classes, not only in the training and testing datasets, but also regarding the amino acid types. The resulting platform and predictor are available at: http://moreiralab/resources/spotone.

## 2. Results

**Figure 1:**
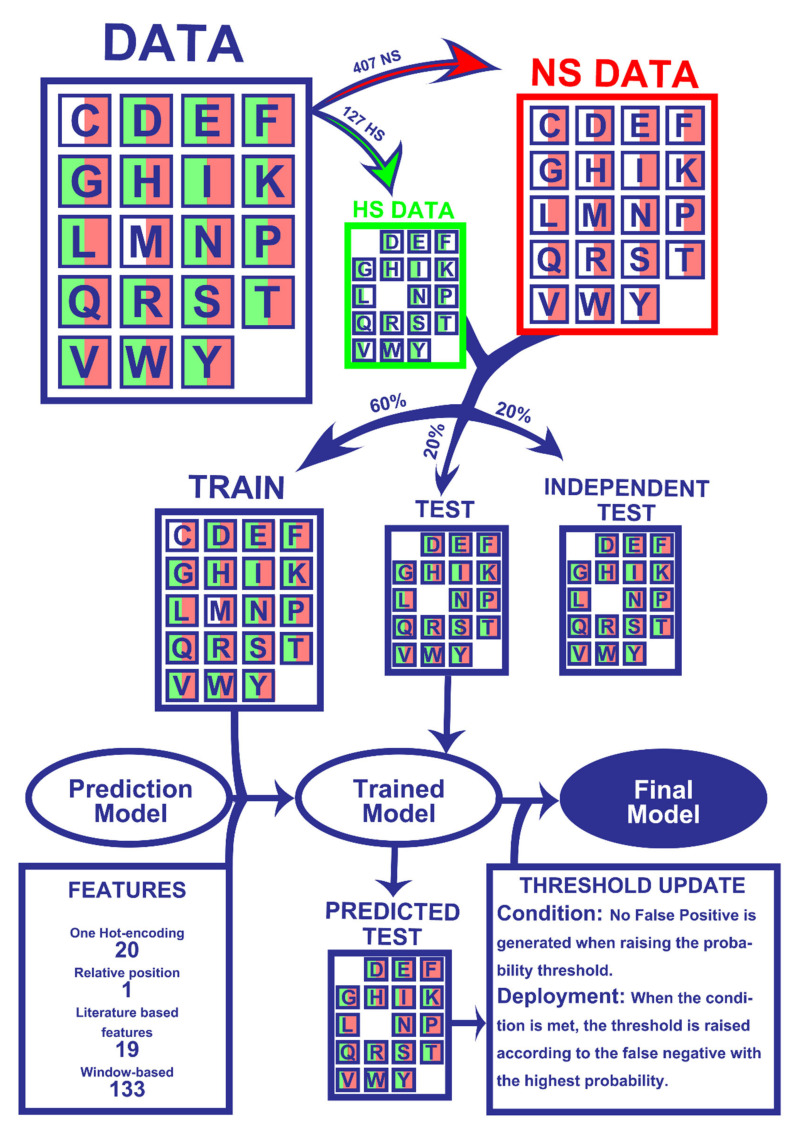
Workflow of the Machine Learning pipeline. Firstly, the 534 amino acids were split into experimentally determined HS (127) and NS (407). Secondly, 60% of the entries of both classes were randomly picked for the train dataset while the remaining 40% were not used for the training phase (20% for test and 20% for an independent test). All datasets were matched with their corresponding 173 features. The training data were used to train the models, which were tested on the test set to yield HS/NS predictions. The predictions were then used to update probability thresholds and generate the final model, which basically consists of the trained model with subsequent HS probability correction. The final model was then applied to an independent test, which did not influence any step of the process, in order to be evaluated. More details on the used method can be found in the Materials and Methods Section.

The results presented herein were attained following a ML pipeline, depicted in Figure 1, which lays the overall steps involved in dataset preparation and prediction model training and refinement. The detailed version of each step is further explored in the Material and Methods Section.

### 2.1. Dataset

**Figure 2:**
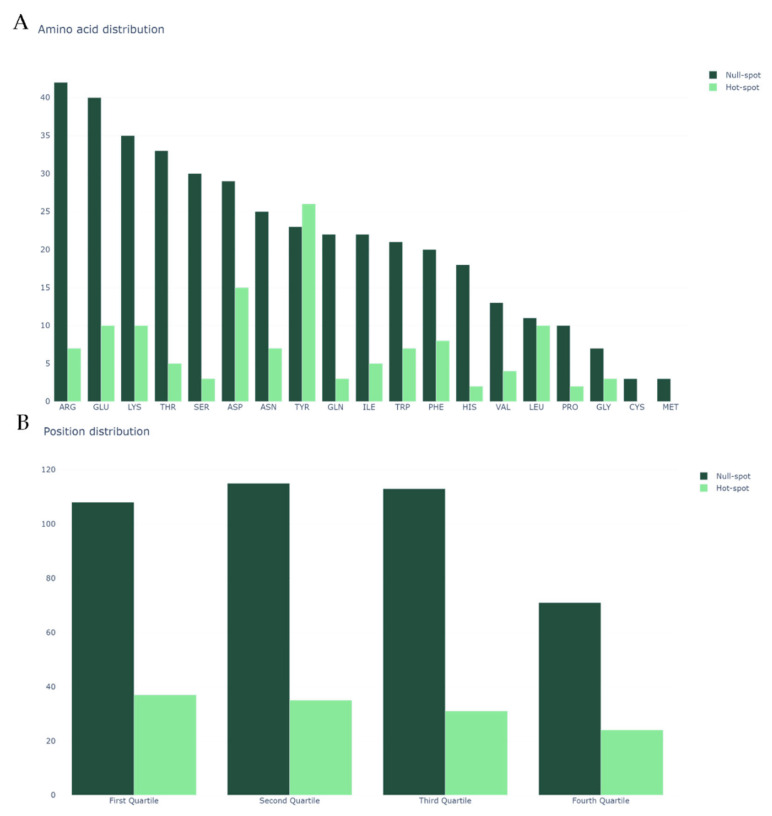
(**A**) Class distribution by amino acid type; and (**B**) class distribution by relative position in the protein sequence. In both plots, the y-axis represents the amino acid count.

We began by analyzing our dataset, the same previously mined and cleaned for SPOTON [22], composed by 534 amino acid residues, of which 127 are HS and 407 are NS, from 53 protein–protein complexes. Figure 2A shows the class distribution by amino acid type. Clearly, TYR, one of the most common HS in nature, is an outlier. Secondly, it should be noted that MET and CYS have no registered HS. Finally, it should also be noted that, due to the nature of the method used for HS experimental determination, there are no ALA residues in either the HS or NS class (as already explained). Figure 2B shows the split of the protein primary sequences into four equally long quartiles, which allowed us to analyze the HS/NS distribution along these ordered sections. It should be noted that, in the first quartile of the protein, the number of HS is at its highest value, although the number of NS is not equally as high. In the last quartile of the protein sequences, the number of overall registered HS/NS is the lowest; however, the proportion in which they stand favors the existence of HS rather than NS, in comparison with the remaining quartiles. The comparison with the literature-based features can be consulted at the landing page of our website. These features include secondary structure propensity, pKa associated values, number of atoms of each type and standard area and mass associated values. Their analyses can show tendencies of these features that correlate to their usefulness to the ML deployment.

### 2.2. Machine-Learning Algorithms

Table A1 and Table A2 in the Appendix A list the full results attained for the various algorithms and methods. Table A1 shows that the in-house built features subset displayed one of the highest performance metrics in comparison with any of the other features alone. It can be noticed that PSSM led to a slight improvement, but the small difference of performance does not compensate the larger amount of time needed for this feature calculation. The introduction of iFeatures, concerning the whole protein, did not increase significantly the performance and introduced concerns related to protein-centered overfitting, and as such was discarded of further studies.

The extremely randomized trees took the lead in most performance metrics, and it is clearly more robust in what concerns the identification of HS, as denoted by the high recall score. It should be noted that neither grid search parameter tuning nor prediction probability tuning according to amino acid type performance was used before method selection to keep the independent test unbiased (further explained in the Material and Methods Section). As such, all values presented in Table 1 concern default settings. This allowed the selection of extremely randomized trees algorithm for parameter tuning, as well as subsequent required alterations.

To avoid the adaptation introduced and displayed in Table A3 leading to the generation of false positives, we set half of the testing set aside, comprising 20% of the whole dataset. Table 2, which lists the performance metrics of the parameter-tuned adapted model for both the training and the testing set, shows a significant increase in the testing performance, while the training scores remain unchanged. This trend was further validated by deploying the model in the independent testing set.

It should be noted that the “class_weight” parameter, available on the deployment of the extremely randomized trees used was particularly relevant in tackling class imbalance, since, by setting it to “balanced_subsample”, it generates and updates class weights based on the samples. A full comparison with state-of-the-art predictions is shown in Table 3. Apart from SPOTON [22], two values for each performance metric are listed: on the left is the value assessed with the dataset used on SPOTONE and on the right are the values presented in the corresponding scientific papers for each method. These values were attained from the pipeline used in SPOTON [22]; since the dataset is the same, the performance comparison also stands equal. In the case of the sequence-based methods that are not SPOTONE, we were not able to deploy our dataset as the webservers indicated were not active or available; this applies to the methods of Nguyen et al. (2013) [19] (reported metrics in their dataset: accuracy of 0.79, recall of 0.59, F1-score of 0.66 and precision of 0.75), Hu et al. (2017) [20] (reported metrics in their dataset: recall of 0.67, F1-score of 0.80 and precision of 1.00) and Liu et al. (2018) [21] (reported F1-score of 0.86 in their dataset).

## 3. Discussion

**Figure A1:**
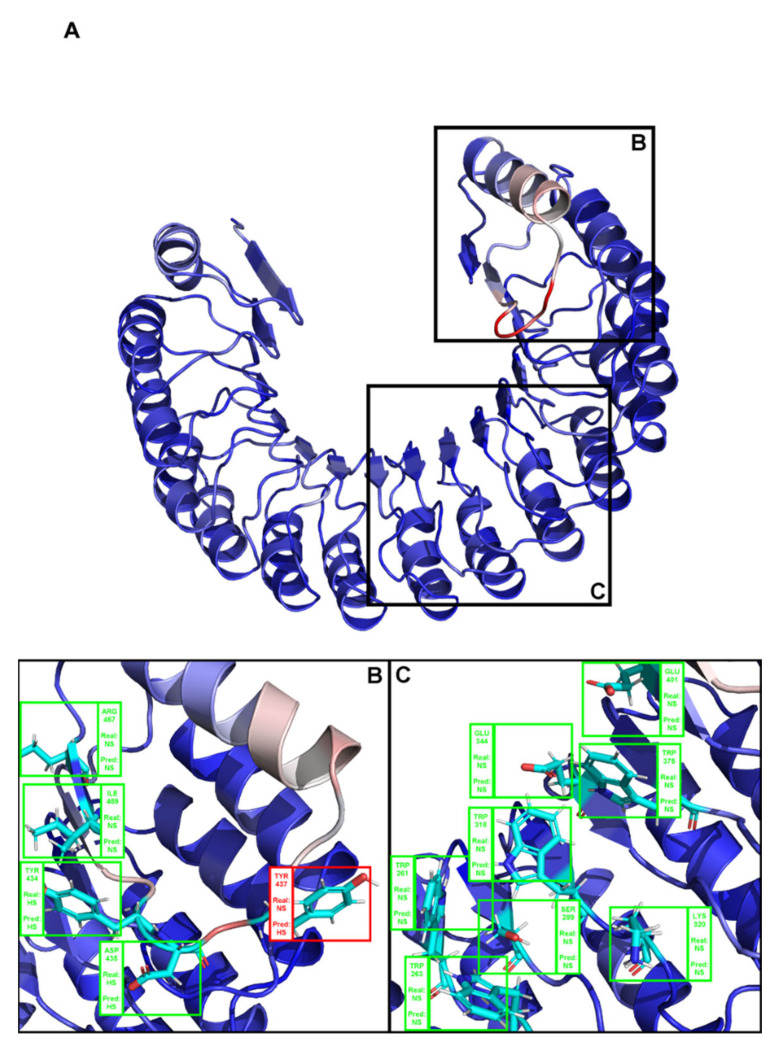
(**A**) Structural representation of the complex between angiogenin and a ribonuclease inhibitor: PDB ID 1a4y. Brighter red colors were attributed to residues with a higher probability of being classified as HS. (**B**,**C**) Close-ins of all interfacial residues for which there is an experimental ΔΔG_binding_ value, and as such a HS/NS classification. Green boxes represent correctly predicted residues, whereas red boxes represent incorrectly classified residues.

**Figure A2:**
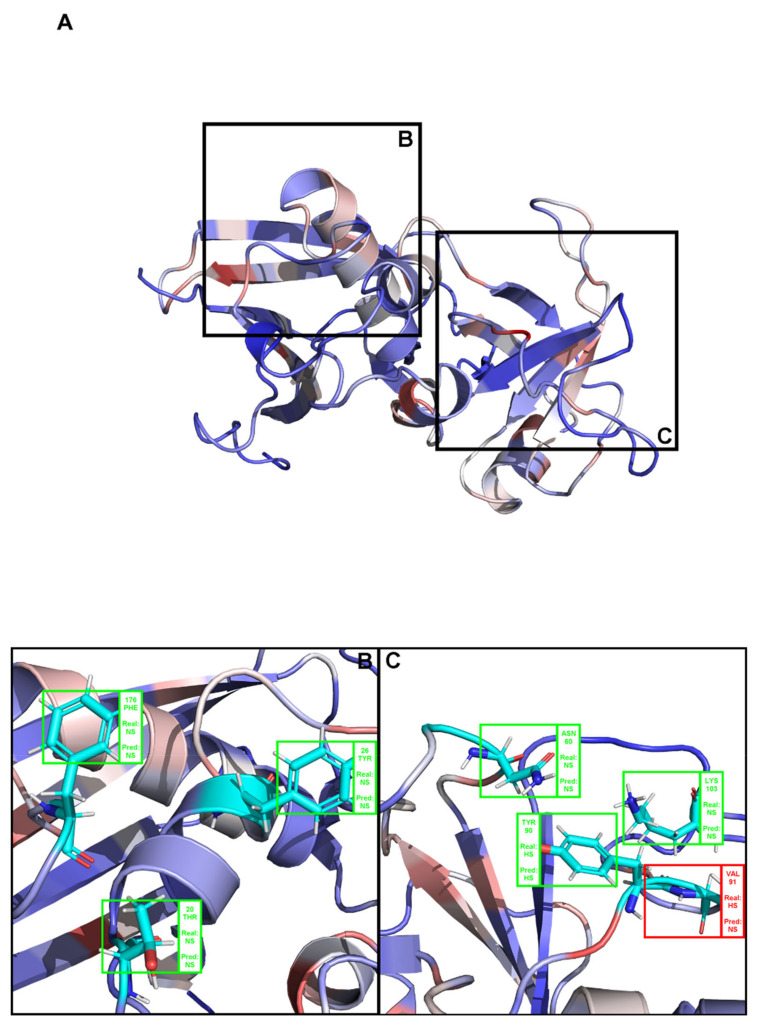
(**A**). Depiction the complex between a T-Cell receptor beta chain and SEC3 superantigen: PDB ID 1jck. Brighter red colors were attributed to residues with a higher probability of being classified as HS. (**B,C**) Close-ins of all interfacial residues for which there is an experimental ΔΔG_binding_ value, and as such a HS/NS classification. Green boxes represent correctly predicted residues, whereas red boxes represent incorrectly classified residues.

**Figure A3:**
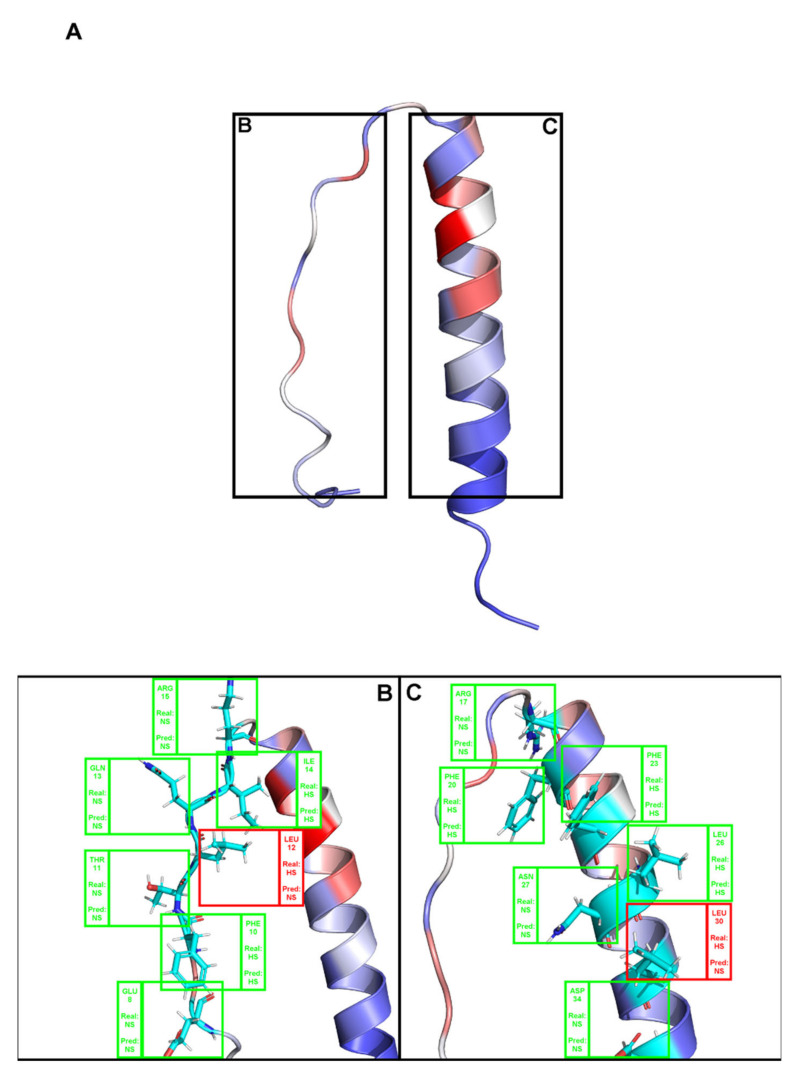
(**A**). Depiction of chain C of the complex PDB ID 3sak. Brighter red colors were attributed to residues with a higher probability of being classified as HS. (**B**,**C**) Close-ins of all interfacial residues for which there is an experimental ΔΔG_binding_ value, and as such a HS/NS classification. Green boxes represent correctly predicted residues, whereas red boxes represent incorrectly classified residues.

This work presents a significant improvement in HS prediction at the interface of protein–protein complexes. However, more than the high performing metrics, the robustness of this model emerges from a thorough treatment and splitting of the dataset, as well as from the exclusion of whole protein sequence features, leaving only residue specific sequence-based features. Figure A1, Figure A2 and Figure A3 display the performance of SPOTONE upon being applied to three different complexes (PDB ids: 1a4y, 1jck and 3sak), with insights on all the residues experimentally determined for these complexes and comparison of this information to our HS/NS SPOTONE prediction. These three examples clearly show how well the predictor works on a point-by-point example. Our final accuracy (0.82), recall (0.82) and precision (0.91) highlight the existence of a very low number of falsely predicted HS as well as NS. Its closeness in performance to the best structural based predictor is complemented with the high versatility of using only sequence-based features prediction, which allows a much wider application in a variety of biological problems.

Finally, all the work is available in a free-to-use platform that allows the user to input one or more protein sequences in FASTA format (Box 1) and attain a detailed HS/NS prediction with corresponding graphical interface. The platform is available at http://moreiralab.com/resources/spotone.

Box 1 **Example FASTA file, with the different proteins’ chains separated by paragraphs and clear identifiers initiated with “>”, separated from the single letter amino acid code chain with a paragraph. This needs to be stored in a “.fasta” file to be submitted to SPOTONE.**

>6Q1G:H|PDBID|CHAIN|SEQUENCE

ASQVQLQESGPGLVKPSGTLSLTCAISGGSISSSNWWTWVRQPPGKGLQWIGEIQHGGGTNYNPSLKSRATIFVDVSKNHFSLRLSSVTAADTAVYYCAKVPPYCTSASCPDDYYYHYMDVWGKGTTVTVSGASTKGPSVFPLAPSSKSTSGGTAALGCLVKDYFPEPVTVSWNSGALTSGVHTFPAVLQSSGLYSLSSVVTVPSSSLGTQTYICNVNHKPSNTKVDKRVEPKSCDKHHHHHH

>6Q1G:L|PDBID|CHAIN|SEQUENCE

ASSSELTQDPAVSVALGQTVRITCQGDSLRGYSASWYQLKPGQAPVLVIYGKNNRPSGIPDRFSGSTSGNRASLIITGTQAEDEADYYCNSRDTNGYRPVLFGGGTKLTVLGQPKGAPSVTLFPPSSEELQANKATLVCLISDFYPGAVTVAWKADSSPVKAGVETTTPSKQSNNKYAASSYLSLTPEQWKSHRSYSCQVTHEGSTVEKTVAPTECS

## 4. Materials and Methods

The dataset used here was retrieved from our previous method, SPOTON [22], and is comprised of 534 amino acid residues (127 positive-HS and 407 negative-NS). This dataset was constructed of data merged from the experimental databases ASEdb [12], BID [13], PINT [14] and SKEMPI [15], and as such comprises all literature available experimental data coming from alanine scanning mutagenesis. We also highlight that sequence redundancy was already eliminated in our previous work. To address this particular problem, we did not simply split the 534 samples into training and testing sets. Firstly, we split all the samples into two datasets containing either HS or NS. Of these datasets, we extracted 20 different subsets from each (corresponding to the 20 possible amino acids). We randomly split these 40 sets (20 HS subsets and 20 NS subsets) in a 60:40 ratio, using “train_test_split” from scikit-learn [28]. Finally, we stitched the tables corresponding to the training set and the testing set back together. Our process was devised to ensure that HS and NS were equally represented for each residue in both the training set and the testing set. Unfortunately, ALA entries were completely absent from the dataset (due to the experimental detection method typically used in wet labs) and CYS and MET only had NS entries (as these residues have a lower/null incidence as key in PPIs). For the latter two cases, we included them in the training set, as it would not be possible to assay their presence in the testing set. Following this procedure, we ended up with a training set containing 312 residues and a testing set containing 222 residues. We randomly split the final testing set in two, with 111 residues each; half the testing set was used to fine-tune probability thresholds (see Prediction Probability Tuning), while the other half was set aside for fully independent test analysis, only having been used after selecting the ML model and performing all parameter tuning.

### 4.1. Features

The following section reports the calculation of 173 features with an in-house Python pipeline and literature-based information on amino acid characteristics. All the extracted features can be calculated simply using the input sequence of a FASTA file. It should be noted that we only used sequence-based features and, furthermore, we did not add any sequence feature about the protein as a “whole”, which might have, due to the size of the dataset, promoted overfitting on a protein level. As shown in Table A1 and Table A2, pre-constructed whole-sequence based features and Position-Specific Scoring Matrix (PSSM) were also tested. For the first, we used iFeature [29] and attained 14.056 whole sequence-based features, for each of the chains. For PSSM, we used an in-house psiblast [30] deployment to extract 42 position conservation features.

### 4.2. One-hot Encoding (20 Features)

The first twenty features extracted for each amino acid residue were simply a one-hot encoded representation of the amino acid; thus, for each amino acid, nineteen columns were filled with “0”, and only one (with the corresponding value), was filled with “1”.

### 4.3. Relative Position Feature (1 Feature)

In Figure 2B, we display the abundance of NS/HS on the protein sequence quartiles. The quartiles were defined by splitting the proteins’ length by four and analyzing the residues present in each of the sections. As such, we used the numbering 1–4 (representing its relative position in the sequence) as a feature that indicates the quartile in which each amino acid is present.

### 4.4. Literature-Based Features (19 Features)

Several amino acid properties are constantly determined, updated and made available online. We downloaded 19 amino acid properties from the BioMagResBank [31] and associated each of them with each of the amino acids; the features and corresponding values per amino acid used are listed in Table A4 and Table A5. Please note that this database is regularly updated to improve the reliability of the experimental data. The statistical distribution of these properties regarding their HS/NS on the dataset used are available in form of violin, scatter and boxplots on the landing page (http://www.moreira.com/resources/spotone).

### 4.5. Window-Based Features (133 Features)

Window-based features were described with a “sliding windows” that stopped on the target residue and considered the residues that stand close to it, sequence wise. We considered window sizes of 2, 5, 7, 10, 25, 50 and 75 amino acid residues, and, for each target residue, averaged the values corresponding to the features of in the Literature-Based Features Section on the residues comprised in the windows. Thus, if we multiply the number of raw features (19) by the number of windows (7), we added 133 features.

### 4.6. Machine-Learning Models Deployment

We exploited different algorithms: Neural Networks (“MLPClassifier”) [32], Random Forest (“RandomForestClassifier”) [33], AdaBoost (“AdaBoostClassifier”), Support Vector Machine (“SVC”) [34] and Extremely Randomized Trees (“ExtraTreesClassifier”) [35]. All of the algorithms were used from their scikit-learn [28] deployment. The extremely randomized trees algorithm, similar to a random forest, is based on decision trees. From the training set, the algorithm picks attributes at random and generates subsets; by training these on the decision trees that comprise the model, an ensemble model is built by majority vote. However, one of the main differences to other algorithms is that it chooses node cut-points (the bifurcation points’ thresholds in a decision tree) fully at random; another significant difference is that the full training set is used, instead of a bootstrap replica, for each of the decision trees that comprise the ensemble model. This additional randomization is ideal in small datasets, in which overfitting is more likely to occur on the training set without a proper test evaluation of robustness. This method has proven to have successful results in solving other biological based problems [36,37]. After running all the methods in default scikit-learn [28] settings, we fine-tuned some parameters of the extremely randomized trees [35] with a grid search (“GridSearchCV”, scikit-learn [28]), and the following parameters were updated: “n_estimators”: 500; “bootstrap”: True; and class_weight: “balanced_subsample”. The full set of parameters can be consulted in Table A6, the parameters not referred were kept as default. Grid search was performed with 10-fold cross-validation.

### 4.7. Model Evaluation

To evaluate the models, we subjected both the training and the testing set to confusion matrix analysis. This table relates the actual and the predicted instances (sample) and compares them by their binary status of Negative (N) or Positive (P) in the prediction to their actual class of True (T) or False (F). It further relates these in four different possible combination states: True Negative (TN) is when the prediction is N and the actual is F; True Positive (TP) is when the prediction is P and the actual is T; False Negative (FN) is when the prediction is N and the actual is T; and False Positive (FP) is when the prediction is P and the actual is F.

The confusion matrix allows the calculation of several metrics, such as accuracy (Equation (1)); precision (Equation (2)); sensitivity, recall or True Positive Rate (recall, Equation (3)); False Positive Rate (FPR, Equation (4)); F1-score (Equation (5)); and Area Under the Receiver Operating Characteristic curve (AUROC, Equation (6)). All these metrics were used from the scikit-learn package ^20^.

(1) $$\mathrm{accuracy}=\;\frac{\mathrm{TP}+\mathrm{TN}}{\mathrm{TP}+\mathrm{TN}+\mathrm{FP}+\mathrm{FN}}$$

(2) $$\mathrm{precision}=\;\frac{\mathrm{TP}}{\mathrm{TP}+\mathrm{FP}}$$

(3) $$\mathrm{recall}=\;\frac{\mathrm{TP}}{\mathrm{TP}+\mathrm{FN}}$$

(4) $$\mathrm{FPR}=\;\frac{\mathrm{FP}}{\mathrm{FP}+\mathrm{TN}}$$

(5) $$F1-\mathrm{score}=\;\frac{2*\left(\mathrm{precision}*\mathrm{recall}\right)}{\left(\mathrm{precision}+\mathrm{recall}\right)}$$

(6) $$\mathrm{AUROC}=\;\int_{x=0}^{1}\mathrm{TPR}\left({\mathrm{FPR}}^{-1}\left(x\right)\right)\mathrm{dx}$$

### 4.8. Prediction Probability Tuning

We performed further inspection of the HS/NS prediction by amino acid, in addition to the whole dataset, as can be seen in the “original” rows in Table A3. This inspection led us to notice that the HS/NS ratio had a significant toll in model performance. For example, TYR had a robust prediction of HS/NS; however, residues which had not such a balanced HS/NS ratio performed more poorly. Although this is a classification problem, most classification methods calculate class probability before yielding the predicted class, which is determined according to the higher probable class. As such, we examined the probability associated to the positive class (HS). Upon inspection of classification probabilities of the actual residues, it was noticed that, although not classified as HS, most of these amino acids still had a higher probability of HS classification than NS. The adaptation value displayed in Table A3 is the increase in probability of the HS class, added post-training, that allows higher HS probability amino acids to reach the HS class (above 50%). This value was implemented following the condition that it should not generate FP while increasing the amount of TP. As such, when, for each amino acid, the maximum false negative HS probability was higher than the maximum true negative HS probability, the HS probability (for that amino acid) was updated (Equation (7)). CYS, MET and ALA were not displayed in Table A3 due to their absence from the testing set.

(7) Correction factor= 0.50 – Maximum False Negative HS probability

### 4.9. Webserver Implementation

**Figure 3:**
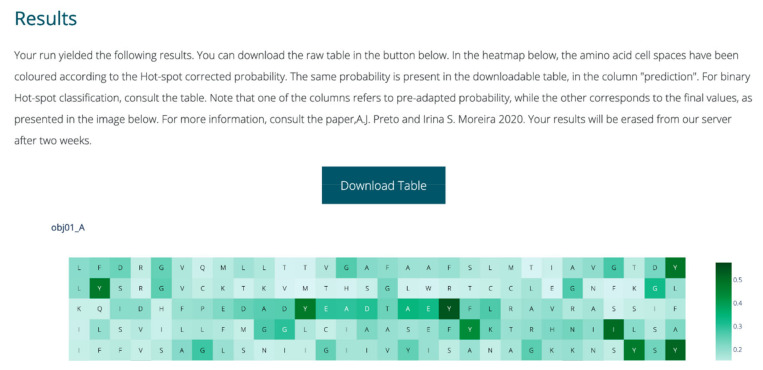
Sample of the output page of SPOTONE.

The webserver was fully implemented with Python. Plotly [38] was used for dynamic graphical representations; scikit-learn [28] was used to perform user submission treatment, analysis and prediction; and in-house Python scripts were used to perform all feature extraction and intermediate steps. Flask was used for overall server set-up and visual layout construction [39]. The output each run includes a dynamic heatmap displaying the probability of HS, for each amino acid in the single or more chains submitted by the user. The full table with the classification probabilities as well as binary class before and after class probability tuning are also available for the user to download. A snapshot of the webserver output is displayed in Figure 3.

## 5. Conclusions

SPOTONE is a thorough prediction algorithm that tackles HS classification in a problem-tailored protocol. The pre-processing and ML steps can be the framework for further protein-based structural biology problems, as are innovating in several processes: (1) by highlighting the importance of protein-based overfitting versus amino acid based features; (2) by providing an answer with a set of simple, replicable, in-house features that make use of freely available information and amino acid position; (3) by considering the evaluation of the amino acid prediction capabilities instead of simply the target features at hand; (4) by attributing specific weights to amino acid types as a way to underline that these are not only features but also subsample spaces of the dataset; (5) by introducing a viable sequence-based HS predictor; and (6) by providing an intuitive and biologically relevant data interpretation tool (HS probability maps). Furthermore, SPOTONE as a webserver (http://moreiralab.com/resources/spotone) is easily usable by non-proficient researchers, with an intuitive framework.

## Figures and Tables

**Table 1:** ML results in the training and testing sets using 5 different algorithms and evaluated using the metrics accuracy (Acc), AUROC, precision (Prec), recall (Rec) and F1-score (F1).

Method	Data	Acc	AUROC	Prec	Rec	F1
Neural network	Train	0.81	0.73	0.81	0.81	0.81
	Test	0.69	0.56	0.72	0.69	0.71
AdaBoost	Train	0.98	0.98	0.98	0.98	0.98
	Test	0.71	0.56	0.77	0.71	0.74
Support Vector Machine	Train	0.77	0.00	1.00	0.77	0.87
	Test	0.76	0.00	1.00	0.76	0.86
Extremely Randomized Trees	Train	0.99	1.00	0.99	0.99	0.99
	Test	0.81	0.77	0.88	0.81	0.83

**Table 2:** Performance metrics on the same training and testing sets after updating the prediction thresholds, and evaluated using the metrics accuracy (Acc), AUROC, precision (Prec), recall (Rec) and F1-score (F1).

Data	Acc	AUROC	Prec	Rec	F1
Training after threshold adaptation	0.99	0.99	0.99	0.99	0.99
Testing after threshold adaptation	0.85	0.88	0.93	0.85	0.87
Independent Testing after threshold adaptation	0.82	0.83	0.91	0.82	0.85

**Table 3:** Structure-based HS prediction performances.

Metrics for Testing-set Evaluation	Structure-Based Methods			
	SPOTON [22]	SBHD2 [23]	KFC-A [24]	KFC-B [25]
AUROC	0.91	0.69/0.69	0.66/--	0.67/-
Recall	0.98	0.70/0.77	0.53/0.85	0.28/0.62
F1-score	0.96	0.62/0.86	0.56/-	0.42/-

## Data Availability

All data and code used to perform the described experiences are available at https://github.com/MoreiraLAB/spotone.

## Appendix A

**Table A1:** Performance metrics (training and testing datasets) for the three studied subsets: with only the in-house features (one-hot encoding, relative position, literature based and window-based features), using only PSSM features and the joint dataset with both in-house and PSSM features.

Dataset	Classifier Name	Subset	Accuracy	AUC	Precision	Recall	F1-Score
In-house features	Extremely Randomized Trees	Train	0.99	1.00	0.99	0.99	0.99
		Test	0.81	0.77	0.88	0.81	0.83
	Neural Network	Train	0.81	0.73	0.81	0.81	0.81
		Test	0.69	0.56	0.72	0.69	0.71
	AdaBoost	Train	0.98	0.98	0.98	0.98	0.98
		Test	0.71	0.56	0.77	0.71	0.74
	Support Vector Machine	Train	0.77	0.00	1.00	0.77	0.87
		Test	0.76	0.00	1.00	0.76	0.86
PSSM features	Extremely Randomized Trees	Train	0.96	0.98	0.97	0.96	0.97
		Test	0.72	0.55	0.82	0.72	0.76
	Neural Network	Train	0.96	0.97	0.96	0.96	0.96
		Test	0.70	0.57	0.74	0.70	0.72
	AdaBoost	Train	0.91	0.92	0.93	0.91	0.91
		Test	0.73	0.60	0.79	0.73	0.75
	Support Vector Machine	Train	0.80	0.86	0.96	0.8	0.86
		Test	0.76	0.64	0.92	0.76	0.82
In-house + PSSM	Extremely Randomized Trees	Train	1.00	1.00	1.00	1.00	1.00
		Test	0.83	0.86	0.93	0.83	0.86
	Neural Network	Train	0.83	0.78	0.85	0.83	0.82
		Test	0.56	0.50	0.52	0.56	0.53
	AdaBoost	Train	0.98	0.98	0.98	0.98	0.98
		Test	0.72	0.60	0.74	0.72	0.73
	Support Vector Machine	Train	0.77	0.00	1.00	0.77	0.87
		Test	0.76	0.00	1.00	0.76	0.86

**Table A2:** Performance metrics for the subsets: The joint dataset with both in-house (one-hot encoding, relative position, literature based and window-based features) and iFeature features (full sequence features); the dataset with in-house, PSSM and iFeature features; and the dataset with only iFeatures.

Dataset	Classifier Name	Subset	Accuracy	AUC	Precision	Recall	F1-Score
In-house + iFeatures	Extremely Randomized Trees	Train	1.00	1.00	1.00	1.00	1.00
		Test	0.83	0.77	0.85	0.83	0.84
	Neural Network	Train	0.77	0.00	1.00	0.77	0.87
		Test	0.76	0.00	1.00	0.76	0.86
	AdaBoost	Train	0.99	0.99	0.99	0.99	0.99
		Test	0.81	0.75	0.83	0.81	0.82
	Support Vector Machine	Train	0.77	0.00	1.00	0.77	0.87
		Test	0.76	0.00	1.00	0.76	0.86
In-house + PSSM + iFeatures	Extremely Randomized Trees	Train	1.00	1.00	1.00	1.00	1.00
		Test	0.83	0.77	0.84	0.83	0.83
	Neural Network	Train	0.77	0.00	1.00	0.77	0.87
		Test	0.76	0.00	1.00	0.76	0.86
	AdaBoost	Train	0.99	0.99	0.99	0.99	0.99
		Test	0.77	0.69	0.79	0.77	0.78
	Support Vector Machine	Train	0.77	0.00	1.00	0.77	0.87
		Test	0.76	0.00	1.00	0.76	0.86
iFeatures	Extremely Randomized Trees	Train	0.83	0.80	0.90	0.83	0.85
		Test	0.77	0.67	0.82	0.77	0.79
	Neural Network	Train	0.77	0.00	1.00	0.77	0.87
		Test	0.76	0.00	1.00	0.76	0.86
	AdaBoost	Train	0.83	0.76	0.86	0.83	0.84
		Test	0.79	0.72	0.80	0.79	0.79
	Support Vector Machine	Train	0.77	0.00	1.00	0.77	0.87
		Test	0.76	0.00	1.00	0.76	0.86

**Table A3:** Extremely randomized trees algorithm scores, by amino acid, in the testing set.

Amino Acid		Adaptation Value	Accuracy	Precision	Recall	Amount Used for Threshold Adaptation
ASP	Original	-	0.71	0.00	1.00	11
	Adapted		-	-	-	
SER	Original	-	1.00	0.00	0.00	4
	Adapted		-	-	-	
GLN	Original	-	0.67	0.00	0.00	6
	Adapted		-	-	-	
LYS	Original	-	1.00	1.00	1.00	12
	Adapted		-	-	-	
ILE	Original	+0.15	0.80	0.00	0.00	5
	Adapted		1.00	1.00	1.00	
PRO	Original	+0.15	0.50	0.00	0.00	2
	Adapted		1.00	1.00	1.00	
THR	Original	-	1.00	1.00	1.00	8
	Adapted		-	-	-	
PHE	Original	+0.25	0.75	0.00	0.00	4
	Adapted		1.00	1.00	1.00	
ASN	Original	+0.15	0.50	0.00	0.00	6
	Adapted		0.83	0.67	1.00	
GLY	Original	-	1.00	0.00	0.00	1
	Adapted		-	-	-	
HIS	Original	-	0.80	0.00	0.00	5
	Adapted		-	-	-	
LEU	Original	+0.06	0.50	0.00	0.00	4
	Adapted		1.00	1.00	1.00	
ARG	Original	-	1.00	0.00	0.00	9
	Adapted		-	-	-	
TRP	Original	-	0.71	0.00	0.00	7
	Adapted		-	-	-	
VAL	Original	+0.25	0.67	0.00	0.00	3
	Adapted		0.67	0.00	0.00	
GLU	Original	-	0.85	0.00	0.00	13
	Adapted		-	-	-	
TYR	Original	-	0.55	0.33	0.67	11
	Adapted		-	-	-	

**Table A4:** Literature-based amino acid features, such as secondary structure propensity, pKa associated values, number of atoms of each type and standard area and mass associated values, attained from BioMagResBank [31].

Amino Acid	Helix Propensity	Sheet Propensity	Helix Propensity Values	Sheet Propensity Values	Molecular Weight	pKa Carboxylate	pKa Amine	pKa Side Chain	Number of Carbons
ALA	1	1	1.45	0.97	89.09	2.30	9.90	0.00	3
CYS	2	2	0.77	1.30	121.16	1.80	10.80	8.65	3
ASP	2	3	0.98	0.80	133.10	2.00	10.00	4.04	4
GLU	1	4	1.53	0.26	147.13	2.20	9.70	4.39	5
PHE	3	2	1.12	1.28	165.19	1.80	9.10	0.00	9
GLY	4	3	0.53	0.81	75.07	2.40	9.80	0.00	2
HIS	3	5	1.24	0.71	155.16	1.80	9.20	6.75	6
ILE	5	6	1.00	1.60	131.17	2.40	9.70	0.00	6
LYS	5	5	1.07	0.74	146.19	2.20	9.20	11.00	6
LEU	1	2	1.34	1.22	131.17	2.40	9.60	0.00	6
MET	3	6	1.20	1.67	149.21	2.30	9.20	0.00	5
ASN	6	5	0.73	0.65	132.12	2.00	8.80	0.00	4
PRO	4	5	0.59	0.62	115.13	2.00	10.60	0.00	5
GLN	3	2	1.17	1.23	146.15	2.20	9.10	0.00	5
ARG	2	3	0.79	0.90	174.20	1.80	9.00	12.50	6
SER	2	5	0.79	0.72	105.09	2.10	9.20	0.00	3
THR	2	2	0.82	1.20	119.12	2.60	10.40	0.00	4
VAL	3	6	1.14	1.65	117.15	2.30	9.60	0.00	5
TRP	3	2	1.14	1.19	204.22	2.40	9.40	0.00	11
TYR	6	2	0.61	1.29	181.19	2.20	9.10	9.75	9

**Table A5:** Literature-based amino acid features, such as secondary structure propensity, pKa associated values, number of atoms of each type and standard area and mass associated values, attained from BioMagResBank [31].

Amino Acid	Number of Hydrogens	Number of Nitrogen Atoms	Number of Oxygens	Number of Sulphur	Standard Free Area	Protein Standard Area	Folded Buried Area	Mean Fractional Area	Residue Mass	Monoisotopic Mass
ALA	7	1	2	0	118.10	31.50	86.60	0.74	71.08	71.04
CYS	7	1	2	1	146.10	13.90	132.30	0.91	103.14	103.01
ASP	7	1	4	0	158.70	60.90	97.80	0.62	115.09	115.03
GLU	9	1	4	0	186.20	72.30	113.90	0.62	129.12	129.04
PHE	11	1	2	0	222.80	28.70	194.10	0.88	147.18	147.07
GLY	5	1	2	0	88.10	25.20	62.90	0.72	57.05	57.02
HIS	9	3	2	0	202.50	46.70	155.80	0.78	137.14	137.06
ILE	13	1	2	0	181.00	23.00	158.00	0.88	113.16	113.08
LYS	14	2	2	0	225.80	110.30	115.50	0.52	128.17	128.10
LEU	13	1	2	0	193.10	29.00	164.10	0.85	113.16	113.08
MET	11	1	2	1	203.40	30.50	172.90	0.85	131.19	131.04
ASN	8	2	3	0	165.50	62.20	103.30	0.63	114.10	114.04
PRO	9	1	2	0	146.80	53.70	92.90	0.64	97.12	97.05
GLN	10	2	3	0	193.20	74.00	119.20	0.62	128.13	128.06
ARG	14	4	2	0	256.00	93.80	162.20	0.64	156.19	156.10
SER	7	1	3	0	129.80	44.20	85.60	0.66	87.08	87.03
THR	9	1	3	0	152.50	46.00	106.50	0.70	101.11	101.05
VAL	11	1	2	0	164.50	23.50	141.00	0.86	99.13	99.07
TRP	12	2	2	0	266.30	41.70	224.60	0.85	186.21	186.08
TYR	11	1	3	0	236.80	59.10	177.70	0.76	163.18	163.06

**Table A6:** Extreme randomized trees parameters tested in the Grid Search.

Parameter	Default Value	Tested Values
n_estimators	100	(50,100,250,500,1000)
boostrap	False	(True, False)
class_weight	None	(None,”balanced_subsample”,”balanced”)
criterion	“gini”	(“gini”,”entropy”)
max_depth	None	(None,1,2,3)
min_samples_split	2	(2,3,4,5)
min_samples_leaf	1	(1,2,3)
max_leaf_nodes	None	(None,1,2,3)
max_samples	None	(None,1,2,5,10)
max_features	“auto”	(“auto”,”sqrt”,”log2”)
min_impurity_decrease	0.0	(0.0, 0.01, 0.001)
min_weight_fraction_leaf	0.0	(0.0, 0.01, 0.001)
